# LLM-IE: a python package for biomedical generative information extraction with large language models

**DOI:** 10.1093/jamiaopen/ooaf012

**Published:** 2025-03-12

**Authors:** Enshuo Hsu, Kirk Roberts

**Affiliations:** McWilliams School of Biomedical Informatics, University of Texas Health Science Center at Houston, Houston, TX 77030, United States; Enterprise Development and Integration, University of Texas MD Anderson Cancer Center, Houston, TX 77030, United States; McWilliams School of Biomedical Informatics, University of Texas Health Science Center at Houston, Houston, TX 77030, United States

**Keywords:** natural language processing, large language models, information extraction, named entity recognition, relation extraction

## Abstract

**Objectives:**

Despite the recent adoption of large language models (LLMs) for biomedical information extraction (IE), challenges in prompt engineering and algorithms persist, with no dedicated software available. To address this, we developed *LLM-IE*: a Python package for building complete IE pipelines.

**Materials and Methods:**

The *LLM-IE* supports named entity recognition, entity attribute extraction, and relation extraction tasks. We benchmarked it on the i2b2 clinical datasets.

**Results:**

The sentence-based prompting algorithm resulted in the best 8-shot performance of over 70% strict F1 for entity extraction and about 60% F1 for entity attribute extraction.

**Discussion:**

We developed a Python package, *LLM-IE,* highlighting (1) an interactive LLM agent to support schema definition and prompt design, (2) state-of-the-art prompting algorithms, and (3) visualization features.

**Conclusion:**

The *LLM-IE* provides essential building blocks for developing robust information extraction pipelines. Future work will aim to expand its features and further optimize computational efficiency.

## Background and significance

The use of large language models (LLMs) for IE in natural language processing (NLP) has gained increasing popularity.^1^ There are several benefits including (1) low annotation requirement through zero-shot and few-shot learning,^2^^,^^3^ (2) comparable performance to fully fine-tuned models on biomedical NLP tasks (eg, medication extraction, medication status classification, and medication attribute extraction),^3^ and (3) end-to-end entity span and relation extraction (RE) which complicated entities and relations can be extracted by the same model in a single step.^4^ In the biomedical field where manually labeled gold standards are expensive and IE schemas are often complex, LLM-based IE methods show great promise. Recent works have been focusing on (1) LLM inferencing infrastructures,^5–10^ (2) LLM prompting algorithms,^3^^,^^4^^,^^11–17^ and (3) prompt engineering.^18^ However, for NLP practitioners, challenges persist as the inference engines are difficult to configure and depend heavily on computing environment. Further, prompt engineering requires experience, domain knowledge, and effort in iterative development. Finally, despite some studies releasing source code, to our knowledge, no software integrates multiple systems and methods and provides a comprehensive toolkit for the LLM-based IE pipeline building (ie, software that processes large amounts of documents by extracting the entities, attributes, and relations into a structured format). Therefore, we developed a Python package, *LLM-IE*, for the biomedical NLP community.

Our work has the following significance:

1. We build an LLM agent (“Prompt Editor”) to help users write and polish prompt templates.
2. We implement popular prompting algorithms published in the biomedical domain and the open domain and provide simple APIs.
3. We provide a uniform interface for different LLM inference engines which avoids the complexity of configuration.
4. We provide visualization features for entity, attribute, and relation visualization.

## Objective

Our primary aim is to publish a user-friendly Python package for IE on the Python Package Index (PyPi) repository and the GitHub repository. The secondary aim is to benchmark it and provide guidance on the best usage.

## Methods

*LLM-IE* is a comprehensive toolkit that provides building blocks for the construction of LLM-based IE pipelines. The package and documentation are available on PyPi (https://pypi.org/project/llm-ie/) and GitHub (https://github.com/daviden1013/llm-ie)

### Building information extraction pipelines with *LLM-IE* APIs

*LLM-IE* covers the life cycle of an NLP IE pipeline: (1) task definition, (2) prompt design, (3) named entity extraction, (4) entity attributes extraction, (5) RE, (6) data management, and (7) visualization.

In the **task definition** and **prompt design** phases, users work closely with the Prompt Editor, which is an LLM agent with access to many pre-stored prompt templates and guidelines. Users choose an IE algorithm (“extractor”) and start chatting with the Prompt Editor via terminal or IPython (eg, Jupyter Notebooks). On the backend, the Prompt Editor analyzes the users’ requests using the relevant templates and prompt-writing guidelines and generates a prompt template with specific task descriptions, schema definition, output format definition, and input placeholders.

The system prompt for the Prompt Editor:

You are an AI assistant specializing in prompt writing and improvement. Your role is to help users refine, rewrite, and generate effective prompts based on guidelines provided…

The chat prompt template includes a placeholder for prompt guidelines and examples:

# Task description

Chat with the user following the prompt guideline below.

# Prompt guideline

{{prompt_guideline}}

**Figure 1.**
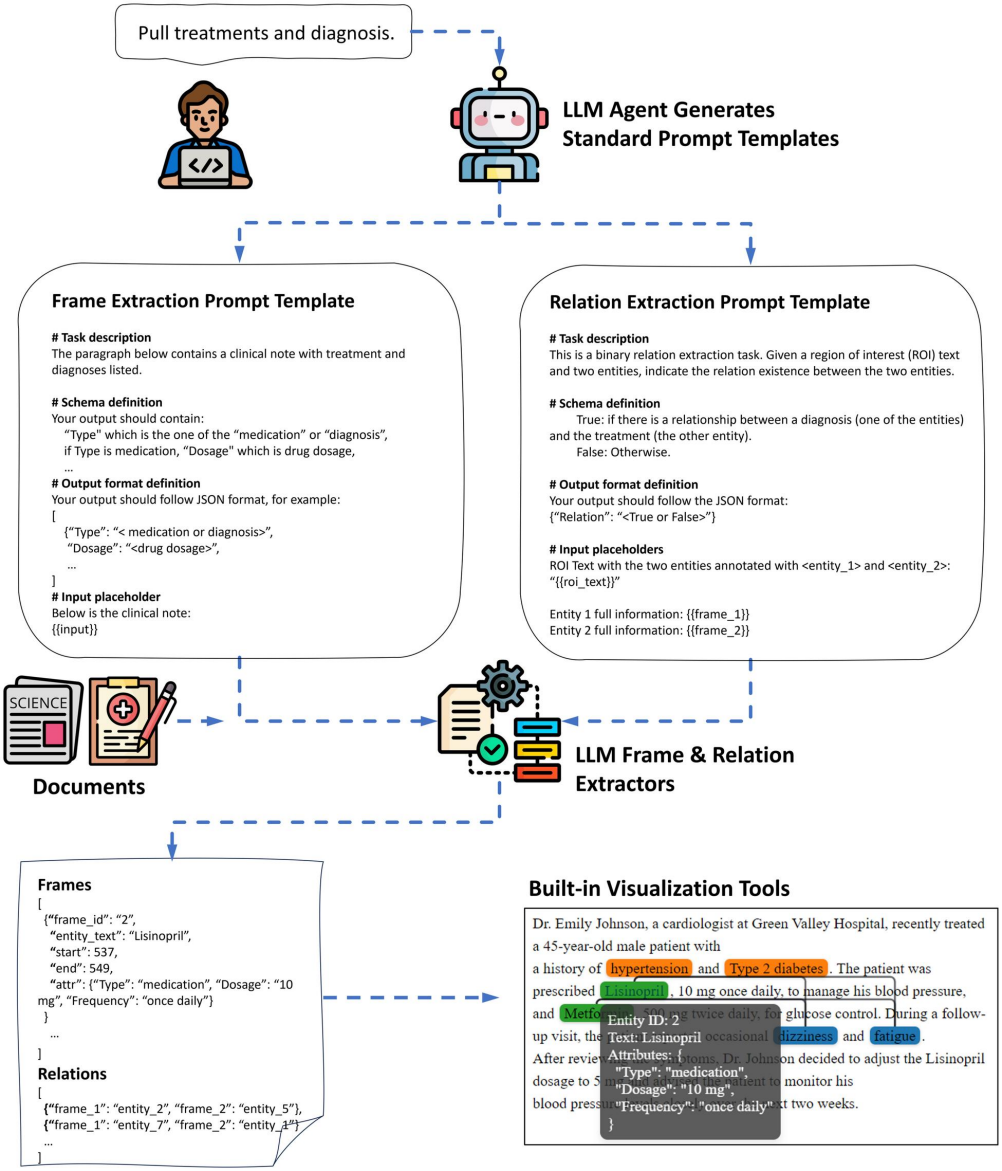
API usage flowchart. Users start by providing a simple description of the task to the LLM agent. The LLM agent generates standard prompt templates with Task description, schema definition, output format definition, and input placeholders. Users iteratively develop prompt templates with the LLM agent until a high-quality prompt template is prepared. The FrameExtractors use the prompt template to extract entities and attributes (“frames”). The RelationExtractors extract the relation and relation types between frames. The built-in visualization tools render the frames and relations on a web App.

Users are encouraged to iteratively develop with the Prompt Editor until a final prompt template is prepared. In the **named entity extraction** and **entity attributes extraction** phases, the frame extractor applies the prompt template for end-to-end entity spans and attribute extraction on the target documents. The LLM outputs strings following the JSON schema specified in the prompt template. A post-processing method then converts them into structured frames with frame ID, entity text, entity spans, and a set of attributes. In general, the Sentence Frame Extractor is more suitable for “dense” tasks in which a document contains many entities, while the Basic Frame Extractor is more efficient for “sparse” tasks with fewer entities. The **relation extraction** phase involves the extracted frames from the previous step and a RE prompt template which can be constructed by working with the Prompt Editor. The relation extractors apply the prompt template on pairs of frames to detect relation existence (ie, binary relations) and relation types (ie, multiclass relations). To reduce computation for LLM inferencing, users are encouraged to provide a pre-processing function (ie, possible_relation_types_func) that applies decision rules. For example, if the 2 frames in the pair are “drug” and “dosage,” the possible relation types are “Dosage-Drug” and “No-relation,” while “dosage” and “dosage” frames must be “No-relation” and thus do not require LLM inferencing. The choice of frame and relation extractors could be empirical (eg, by running a pilot dataset) or experimental (eg, by evaluating validation sets). After extraction, users are recommended to apply the built-in data types (eg, LLMInformationExtractionDocument) for **data management** and further **visualization** through a built-in Flask App or HTML rendering function (Figure 1).

### Package design and architecture

Our architectural design follows four principles: (1) **Efficiency**, in which recent and successful inference engines and prompting algorithms are supported (eg, Ollama,^5^ HuggingFace-hub,^19^ Llama.cpp,^6^ vLLM,^7^ OpenAI API). (2) **Flexibility**, in which fundamental functions are implemented as modules and classes (eg, Inference Engines, Frame Extractors, Relation Extractors) for easy customization. (3) **Transparency**, in which all the prompt templates, LLM inputs, and outputs are accessible to users. (4) **Minimalism**, in which the package has few dependencies. Users only install dependencies for functions they use. This section breaks down the internal modules following the order of dependencies.

**Figure 2.**
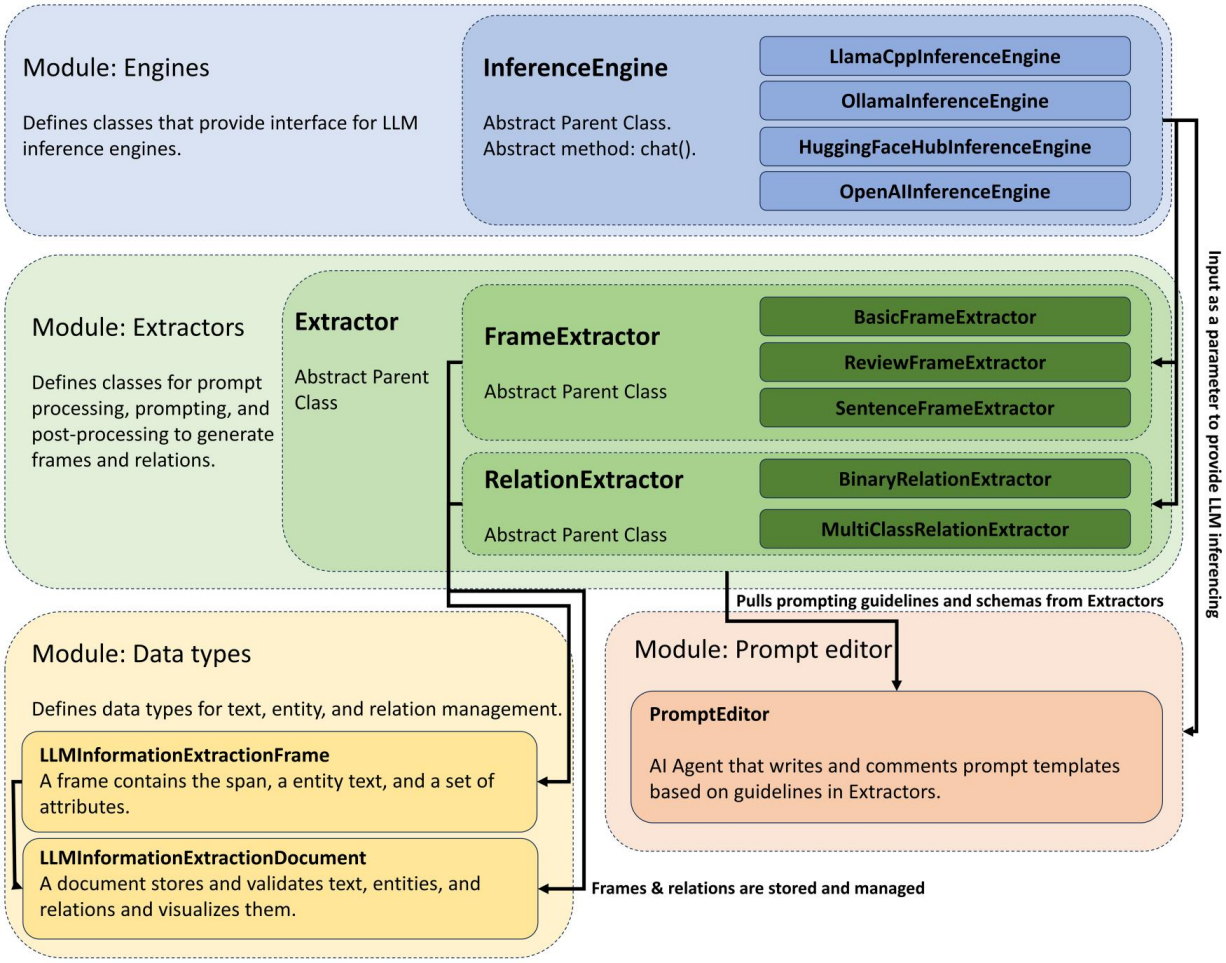
Conceptual class diagram. The Engines module defines InferenceEngine classes that host LLM and provides an interface for inference. The Extractors module defines FrameExtractors and RelationExtractors that process and apply prompt templates, prompt LLM for information extraction, and post-process outputs. The PromptEditor module defines a PromptEditor LLM Agent to chat, write, and comment on prompt templates. The data types module defines containers for text, entities, and relations management and visualization.

The LLM-IE package is composed of four Python modules: (1) Engines, (2) Extractors, (3) Prompt editor, and (4) Data types. The **Engines module** defines interface classes that support popular open-source (eg, Ollama, HuggingFace-hub) and closed-source (eg, OpenAI API) LLM inference engines. They work for the Prompt Editor and extractors. The **Extractors module** defines prompting algorithms (“extractors”) for frame and RE. The Basic frame extractor prompts LLM directly and outputs a list of frames. The Review frame extractor prompts LLM to generate initial outputs and prompt again for amendment and correction. The Sentence frame extractor splits the target document into sentences and prompts sentence by sentence to improve recall and entity span detection accuracy. The binary relation extractor prompts LLM to review and detect relations between a pair of frames. The multi-class relation extractor prompts LLM to classify relation types between a pair of frames. The algorithm sources are summarized in Table 1. The **Prompt editor module** defines a Prompt Editor class that serves as an LLM agent for prompt development. It has access to pre-stored prompt-writing guidelines and examples for each extractor. The **Data types module** defines data management classes for frames and relations storage, validation, and visualization. A document is packaged into a self-contained object. The validation checks for overlaps and redundancy and ensures that relations are linking two existing frames. For minimalism, we implemented the visualization methods (ie, viz_serve, viz_render) by internally calling our plug-in Python package, “ie-viz” (Figure 2).

**Table 1.** Prompting algorithm sources.

Task	Algorithms (implemented in extractors)	Algorithm references
NER	BasicFrameExtractor	^3^
	ReviewFrameExtractor	^11^ ^,^ ^12^
	SentenceFrameExtractor	^13^ ^,^ ^14^ ^,^ ^20^
Entity attribute extraction	All above FrameExtractors	^4^
RE	BinaryRelationExtractor	^15^ ^,^ ^16^
	MultiClassRelationExtractor	^15–17^

### Benchmarking

We benchmarked our package on three clinical NLP datasets for named entity recognition (NER), entity attribute extraction (EA), and RE. We adopted the 2012^21^ and 2014^22^ Integrating Biology and the Bedside (i2b2), and 2018 National NLP Clinical Challenges (n2c2)^23^ Natural Language Processing Challenge. All experiments were evaluated with the Llama-3.1-70B^24^ in an 8-shot prompting setting and conducted with the vLLM^7^ inference engine on a GPU server with 8 NVIDIA A100 GPUs. Details and source code are discussed on our GitHub page (https://github.com/daviden1013/LLM-IE_Benchmark).

The i2b2/n2c2 data user agreement prohibits public sharing of the text content. Therefore, we synthesized clinical notes to demonstrate the final results and visualization. The task is to extract drugs, conditions, and adverse drug events (ADEs) with corresponding attributes and relations. Implementation details are available on our GitHub page (https://github.com/daviden1013/LLM-IE_Benchmark).

## Results

### Benchmarking

**Figure 3.**
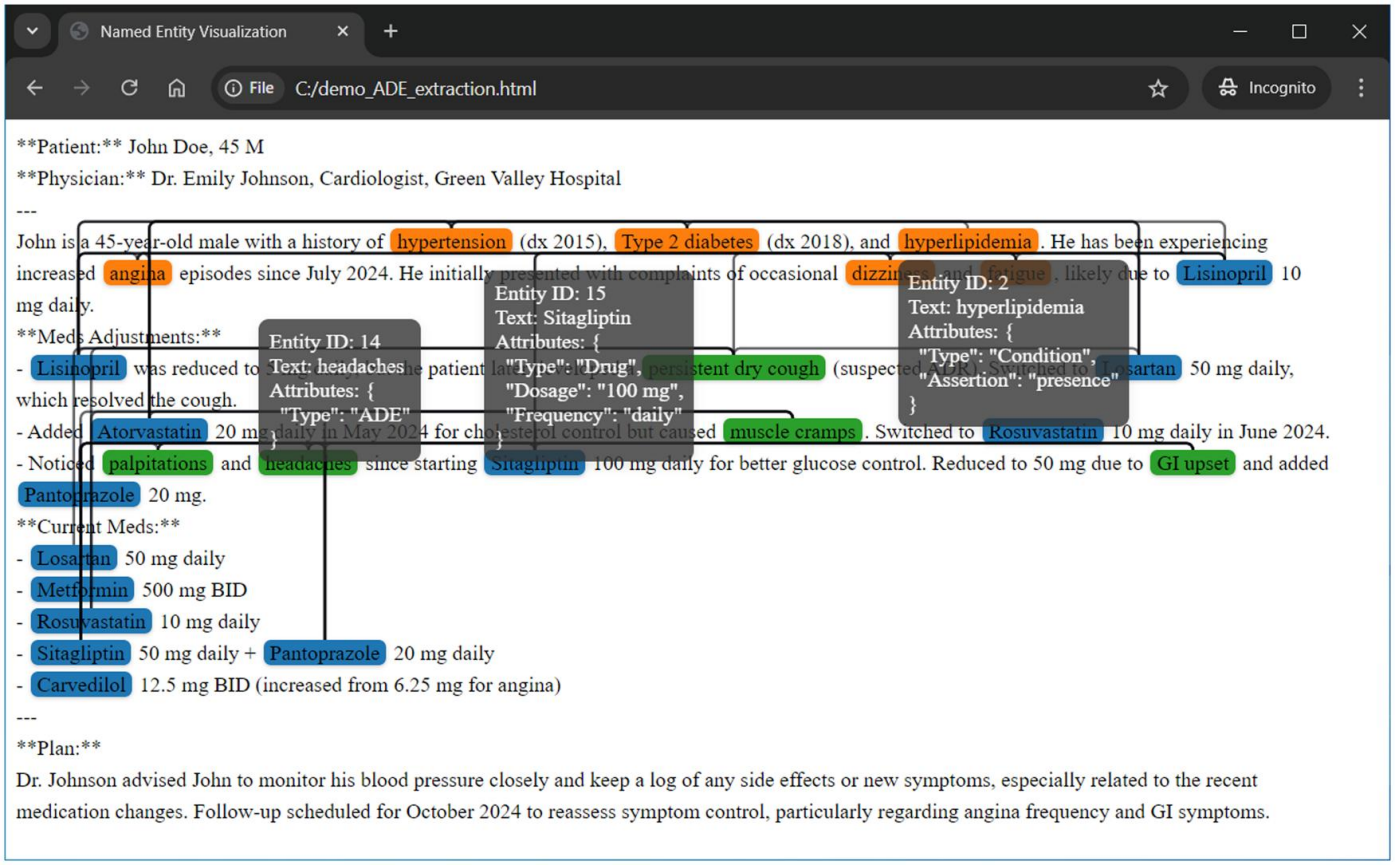
Visualization of a synthesized clinical note. The frames are highlighted based on the attribute “Type” as Drug, Condition, or ADE. For the Drug frames, attributes “Dosage” and “Frequency” are extracted. For the Condition frames, the attribute “Assertion” is extracted. The relations Condition-Drug and ADE-Drug are visualized as paths. Note that for publication purposes, only a few entity attributes (tooltips) are displayed in this figure.

For the NER and EA tasks, the Sentence Frame Extractor achieved the best F1 scores (>0.701 for NER tasks, ∼0.600 for most AE tasks), while consuming more GPU time (up to 2 minutes per note). The Review Frame Extractor had higher recall than the Basic Frame Extractor on all NER tasks. For the RE tasks, the multi-class Extractor achieved high recall (0.978). However, the precision was lower (0.3831) (Table 2). On the synthesized clinical note, the drugs, conditions, and ADE frames and relations were extracted and visualized in Figure 3.

**Table 2.** Benchmark on the i2b2/n2c2 datasets for NER, EA, and RE tasks.

Tasks	Algorithm	GPU time (s)/note	Benchmarks					
**NER**			**2012 temporal relations challenge**					
			EVENT			TIMEX		
			Precision	Recall	F1	Precision	Recall	F1
	Basic	67.5	0.9406	0.2841	0.4364	0.9595	0.3516	0.5147
	Review	84.0	0.8965	0.3995	0.5527	0.9352	0.5473	0.6905
	Sentence	132.9	0.9101	0.6824	0.7799	0.8891	0.739	0.8071

			**2014 de-identification challenge**					
			Strict			Relaxed		
			Precision	Recall	F1	Precision	Recall	F1
	Basic	9.4	0.7154	0.4813	0.5755	0.7172	0.4826	0.5769
	Review	15.7	0.5649	0.5454	0.555	0.5667	0.5471	0.5567
	Sentence	20.7	0.6683	0.7379	0.7014	0.6703	0.7401	0.7035

			**2018 (Track 2) ADE and medication extraction challenge**					

			Strict			Lenient		
			Precision	Recall	F1	Precision	Recall	F1
	Basic	44.3	0.7384	0.3534	0.478	0.8537	0.4034	0.5479
	Review	63.2	0.7209	0.427	0.5363	0.8416	0.4918	0.6208
	Sentence	114.1	0.852	0.6166	0.7154	0.963	0.692	0.8053

**Entity attribute extraction**			**2012 temporal relations challenge**					
			EVENT			TIMEX		
			Type	Polarity	Modality	Type	Value	Modifier
	Basic	67.5	0.2589	0.2707	0.2737	0.3236	0.2835	0.3198
	Review	84.0	0.358	0.3799	0.3828	0.4934	0.4209	0.4857
	Sentence	132.9	0.6056	0.642	0.6432	0.678	0.5505	0.667
	
**RE**			**2018 (Track 2) ADE and medication extraction challenge**					
			Precision		Recall		F1	
	Multi-class	213.9	0.3831		0.978		0.5505	

## Discussion

We developed the *LLM-IE* Python package for LLM-based IE. The usage (ie, building block classes and pipelines) is designed based on our practical NLP experience in the healthcare industry. We have been adopting it internally for NLP projects. Therefore, we believe it is relevant to other NLP practitioners in the biomedical field. The architectural design in which inference engines and extractors are placed in modules with well-organized inherent relationships enables continuous development as new infrastructures and prompting algorithms are released in the future. Our visualization features provide an intuitive way to validate (eg, error analysis, performance evaluation) outputs with a complex schema which would be cumbersome otherwise.

The benchmark results are reasonable compared to our recent publication.^25^ Compared with fully supervised systems, the average lenient F1 score of 2018 n2c2 participants was 0.8051.^23^ Our Sentence Frame Extractor achieved a comparable result (0.8053) while only using 8 sentences (“8-shot”). However, in some other tasks, the few-shot LLM performance was below fully supervised models, as previously reported.^15^ Further development (eg, prompt engineering and error analysis) is needed to improve the performance.

Despite the great features, our *LLM-IE* package has a few limitations: (1) it is in an active development phase. More practical adoption and evaluation are needed. (2) Like all LLM-based systems, prompt engineering plays an important role in providing domain knowledge and task-specific definitions. Despite our Prompt Editor LLM agent, it is up to the users to finalize the prompt templates. Some familiarity with prompt writing is still necessary. (3) The post-processing relies on the LLM to output in the correct format. Inconsistent elements in the JSON list are discarded. Thus, it is important to choose LLMs with good instruction-following capacity. (4) Our benchmarking used Llama 3.1 to represent the state-of-the-art open-source LLM at this point. Further evaluation is needed for other LLMs.

Our short-term development goals are improving computational performance (eg, concurrent extraction) and optimizing post-processing (eg, automatically fixing inconsistent JSON output formats). In the long term, we aim to implement cutting-edge prompting algorithms and extend support for emerging inference engines.

## Conclusions

To fill in the gaps between the latest LLM technology and biomedical NLP practices, we developed a Python package, *LLM-IE*, that provides building blocks for robust IE pipeline construction.

## Data Availability

The benchmark datasets used in this study are publicly available. Registration is required via the **DBMI portal** (https://portal.dbmi.hms.harvard.edu/). Once approved, dataset requests can be made through the **n2c2 NLP Research Data Sets** webpage (https://portal.dbmi.hms.harvard.edu/projects/n2c2-nlp/). The source datasets are managed by the Department of Biomedical Informatics, Harvard Medical School, 10 Shattuck Street, Suite 514, Boston, MA 02115, Phone (617) 432-2144, fax (617) 432-0693. For the curated research datasets, please contact the corresponding author, Kirk Roberts, kirk.roberts@uth.tmc.edu
